# Determination of Polar Heterocyclic Aromatic Amines in Meat Thermally Treated in a Roasting Bag with Dried Fruits

**DOI:** 10.3390/foods14040559

**Published:** 2025-02-08

**Authors:** Sylwia Bulanda, Magdalena Szumska, Agnieszka Nowak, Beata Janoszka, Aleksandra Damasiewicz-Bodzek

**Affiliations:** Department of Chemistry, Faculty of Medical Sciences in Zabrze, Medical University of Silesia in Katowice, Jordana 19, 41-808 Zabrze, Poland; bulanda.sylwia@gmail.com (S.B.); mszumska@sum.edu.pl (M.S.); agnieszkanowak@sum.edu.pl (A.N.); aleksandra.bodzek@sum.edu.pl (A.D.-B.)

**Keywords:** food carcinogens, roasted meat, apricots, cranberries, prunes, inhibition, liquid chromatography, diode array detection, fluorescence detection

## Abstract

Frequent consumption of processed meat has been classified as carcinogenic to humans by the International Agency for Research on Cancer (Group 1), while red meat has been classified as probably carcinogenic (Group 2A). Mutagenic and carcinogenic compounds formed by heating in protein-rich food include, among others, heterocyclic aromatic amines (HAAs). Modifying the heat treatment of meat and using natural additives with antioxidant properties can lead to a reduction in their formation. The aim of this study was to determine polar HAAs (imidazoquinolines, IQ and MeIQ; imidazoquinoxalines, 8-MeIQx and 4,8-DiMeIQx; and phenylimidazopyridine, PhIP) in pork loin prepared without additives and with three types of dried fruit (apricots, cranberries, and prunes), baked in a roasting bag. HAAs were isolated from meat samples by solid-phase extraction. Quantitative analysis was performed by high-performance liquid chromatography with fluorescence detection (FLD) and a diode array detector (DAD). Only two HAAs, 8-MeIQx and PhIP, were detected in extracts isolated from meat samples. The total content of these compounds in meat roasted without additives was 5.9 ng/g. Using a dried fruit stuffing content of 200 g/kg of meat reduced these concentrations in dishes prepared with prunes, apricots, and cranberries by 42%, 47%, and 77%, respectively.

## 1. Introduction

According to the World Health Organization (WHO) report on the health impact of red and processed meat consumption, the intake of all types of meat has increased significantly worldwide over the last 50 years, with an anticipated additional 50% increase by 2050 [1]. Current WHO health recommendations suggest that red meat consumption for adults should range from 98 g to 500 g per week, equivalent to 26 kg annually [1]. In countries within the Organization for Economic Cooperation and Development (OECD), average meat consumption was recorded at 69.5 kg/person in 2019–2021, although global consumption is nearly half this amount. A calculated 14.3% reduction in red and processed meat consumption, paired with an increase in plant-based food intake, could reduce deaths by approximately 65,000 in countries with the highest consumption of processed meat products [1].

In 2015, the International Agency for Research on Cancer (IARC, Lyon, France) classified red meat as “probably carcinogenic to humans” (Group 2A) based on limited evidence of its association with colorectal cancer in humans and sufficient evidence from animal studies. Processed meat, encompassing smoked, cured, or salted products, was classified as “carcinogenic to humans” (Group 1), based on sufficient evidence linking its consumption to colorectal cancer [2]. During meat heat treatment, compounds with mutagenic and carcinogenic effects may form [3,4] including heterocyclic aromatic amines (HAAs) [5,6,7]. While often, intake of processed meat has been categorized as a carcinogenic factor, it remains a rich and important source of nutrients, such as amino acids, proteins, iron, micronutrients, and B-group vitamins (e.g., B6, B12, and folic acid), along with other bioactive compounds [8]. Due to its significant nutritional value, meat should not be entirely eliminated from diets. However, it is advisable to apply cooking and preparation methods that minimize the amount of compounds hazardous to human health in meat products.

Heterocyclic aromatic amines are typically formed at temperatures used for cooking, frying, roasting, and grilling. The higher the temperature and the longer the heat treatment, the more HAAs are produced in terms of concentration and variety [9,10]. To date, the structures of more than 30 HAAs have been identified [11,12]. Heterocyclic aromatic amines are nitrogen compounds consisting of two or three condensed rings. One of them is aromatic, while the rest are heterocyclic. Aside from three HAAs, all the others contain an exocyclic amino group (-NH_2_) [12,13,14]. At temperatures commonly used for meat preparation (i.e., 100 °C to 250 °C), primarily polar HAAs are formed. These include imidazoquinoline (IQ and MeIQ), imidazoquinoxaline (8-MeIQx and 4,8-DiMeIQx), and imidazopyridine (PhIP) derivatives, known as thermic amines [6]. Their formulas and names are presented in Table 1. The IARC classified the IQ as Group 2A, i.e., probably carcinogenic to humans, and MeIQ, PhIP, and 8-MeIQx as Group 2B, i.e., possibly carcinogenic [15].

Higher temperatures result in the formation of pyridoimidazole and pyridoindole derivatives, generally called carbolines. Small amounts of these pyrolytic, nonpolar HAAs are also found in fried and grilled foods [11]. Polar HAAs form during the multi-stage Maillard reaction from compounds that naturally occur in food, i.e., sugars (glucose, ribose, and fructose), α-amino acids, and creatine [6,12,16]. Initially, amino acid amino groups and sugar carbonyl groups interact to form pyridine, N,N-dialkylpyrazine, and aldehydes. These compounds react with creatinine, which originates from creatine via water elimination and cyclization, forming imidazoquinoxalines and imidazoquinolines. PhIP, an imidazopyridine, may form without sugars, directly from creatinine and certain amino acids (e.g., phenylalanine, tyrosine, and leucine) [6]. During the Maillard reaction, pyrazine, and pyrimidine radicals can form. The synthesis mechanism of polar HAAs likely involves reactions with free radicals and carbonyl compounds [13].

The formation of HAAs and strategies to reduce exposure to these carcinogens have been reviewed extensively [6,7,17,18,19,20,21]. Effective HAA reduction methods include marinating meat in beer, wine, or natural extracts (e.g., tea), especially with spices like oregano, basil, and hawthorn extract [17,22,23]. Studies confirm that plant additives can modify HAA synthesis pathways during meat heat treatment [13,14,24,25]. These additives include spices with high antioxidant properties, such as paprika, ginger, and black pepper [26]. A meta-analysis of studies from 1997 to 2019 revealed that garlic, onion, pepper, and spices with phenolic compounds inhibit HAA formation in fried, grilled, and baked foods. Antioxidants in these additives donate electrons that neutralize free radicals, reducing radical HAA formation processes [19]. Model studies using standard substances showed that antioxidant compounds inhibit multiple Maillard reaction pathways. They prevent HAA formation by quenching free radicals [21]. Phenolic compounds may react with reactive carbonyls formed during food heating [13], which are substrates for synthesizing heterocyclic amines, including imidazoquinolines and imidazoquinoxalines. Lower levels of carbonyl compounds result in fewer HAAs. Studies have also shown that individual phenolic compounds have a positive effect on capturing free radicals and mitigating HAAs. However, for mixed phenolic compounds, the correlation between radical scavenging activity and the inhibitory effect on HAAs was weak [27]. Some studies observed increased HAA levels, especially pyrolytic HAAs like β-carbolines (harman and norharman), when spices and plant additives were used [12,27,28,29]. HAA formation depends on multiple factors, such as cooking temperature; time; method; meat type; and its amino acid, sugar, creatine, and fat content [6,12,30]. Thus, increased HAAs might be due to factors other than the additive type.

Food HAA levels are typically low, at ng/g of meat. Using additives often reduces these levels. Advanced analytical techniques are now employed to detect such compounds, with liquid chromatography coupled with mass spectrometry (LC-MS) or ultra-performance liquid chromatography–tandem mass spectrometry (UPLC–MS/MS) becoming more common [29,30,31]. However, due to high costs of LC-MS systems, HPLC with UV-VIS, diode array (DAD), or fluorescence detectors (FLD) remains in use [27,32,33]. HAA isolation from food often follows modifications of the clean-up method by Gross and Grüter [34]. It involves liquid extraction using diatomaceous earth (Extrelut) with dichloromethane or ethyl acetate as organic solvents and extraction to the solid phase (SPE) on columns packed with an cation exchanger, i.e., propylsulfonic acid (PRS) and a chemically bound C-18 phase to obtain nonpolar and polar fractions of HAAs [28,29,34]. This SPE tandem is sometimes replaced by mixed-mode cation exchange cartridges to trap HAAs in a single fraction [6,35]. In recent years, other extraction procedures such as microwave-assisted extraction and microextraction, including dispersive liquid–liquid microextraction, have gained popularity because of its advantages, with solvent savings and rapid preparation of extracts [32].

Polish cuisine traditionally includes meat dishes roasted or stuffed with fresh or dried fruits. Today, many cookbooks and culinary programs combine this tradition with modern methods, such as roasting meat in electric ovens using roasting bags. This convenient and flavorful method also preserves beneficial taste properties. To the best of our knowledge no studies to date have explored the effect of whole dried fruits on polar HAA occurrence in meat dishes. So far, there have been few publications on the impact of fruit on the formation of HAAs. In contrast to our studies, they focused on plant-based additives (e.g., juices) or fruit extracts and their potential to reduce the concentration of these compounds in meat products [23,36,37,38,39,40,41,42]. Therefore, this study examined selected HAAs (IQ, MeIQ, 8-MeIQx, 4,8-DiMeIQx, and PhIP) in meat roasted with whole dried fruit (prunes, apricots, and cranberries) and assessed whether this preparation method lowered harmful compound levels in the resulting meat products.

## 2. Materials and Methods

### 2.1. Chemicals and Reagents

The following heterocyclic aromatic amines were purchased from Toronto Research Chemicals (Vaughan, ON, Canada): 2-amino-1-methyl-6-phenylimidazo[4,5-b]pyridine (PhIP); 2-amino-3,8-dimethylimidazo[4,5-f]quinoxaline (8-MeIQx); 2-amino-3,4,8-trimethylimidazo[4,5-f]quinoxaline (4,8-DiMeIQx); 2-amino-3,4- dimethylimidazo[4,5-f]quinoline (MeIQ); and 2-amino-3-methylimidazo[4,5-f]quinoline (IQ). The structures of these compounds are shown in Table 1.

The organic solvents—toluene, dichloromethane, and n-hexane (analytical-reagent grade), acetonitrile, and methanol (HPLC-grade)—were from Avantor™ Performance Materials (Gliwice, Poland). The deionized water was obtained from a deionization system (Millipore, Vienna, Austria). The 85% phosphoric acid (V) was obtained from Merck (Darmstadt, Germany), and triethylamine (HPLC grade) was obtained from Fisher Scientific (Leicester, UK). Sodium hydroxide, ammonium acetate, hydrochloric acid, and 25% ammonia solution (analytical-reagent grade) were purchased from Avantor™ Performance Materials (Gliwice, Poland).

Columns with diatomaceous earth (Extrelut NT20) were purchased from Merck (Darmstadt, Germany). Columns for solid-phase extraction (SPE) containing octadecylsilane (SPE-C18, 3 mL, 500 mg) and propylsulfonic acid (SPE-PRS, 3 mL, 500 mg) were from J.T. Baker (Avantor™ Performance Materials BV, Deventer, The Netherlands).

### 2.2. Apparatus

For homogenization with alkaline hydrolysis of meat samples, a homogenizer (MPW-120) from Med. Instruments (Warsaw, Poland) was used. An evaporation system under nitrogen (VLM GmbH, Bielefeld, Germany) was used for solvent evaporation.

A high-performance liquid chromatography (HPLC) system, the Ultimate 3000 TSL from Dionex Softron (Germering, Germany), was used for analyzing HAAs. It was equipped with a thermostatted autosampler (WPS-3000 TSL, Dionex Softron, Germering, Germany) and a column compartment (TCC-3200, Dionex Softron). The system was connected to a diode array detector (DAD) (PDA 3000, Germering, Germany) and a fluorescence detector (FLD) (Shimadzu RF-2000, Kyoto, Japan). Chromeleon software (6.80 SP2 Build 2284, Dionex Softron, Germering, Germany) was used for setting analytical parameters and collecting data.

### 2.3. Meat Preparation

The study samples consisted of pork loin stuffed with dried fruits, prunes, cranberries, and apricots, roasted in an electric oven with roasting bags. Meats such as poultry, game, beef, and pork stuffed with various fruits, dried or fresh (e.g., apples, pears, peaches, cranberries, and prunes), and heat-treated are traditional in Polish cuisine. The Supplementary Materials provides additional information on the dried fruits used in this study.

Two pork loins purchased from a local butcher were used for the study. The meat came from one animal. The bones and fat were removed before preparing the dishes. The meat was cut into four pieces. Each weighed about 1 kg. Cuts were made in three portions to create a hole with a diameter of about 3 cm inside the tenderloin. Each was stuffed with ground dried fruit in the amount of 200 g per 1 kg of pork. Three types of stuffed pork loin (with prunes, apricots, and cranberries) were prepared. The fourth portion, containing no additives, served as the control. Each meat portion, approximately 20 cm long and 10 cm wide, was individually wrapped in aluminum foil and stored in the refrigerator. After 12 h, the foil was removed and each portion was placed in a separate roasting foil so that the meat touched the inner surface of the bag only on the underside. The baking bag was closed tightly on both sides with appropriate clips. Each portion of meat prepared in this way was placed on a roasting tray in an electric oven preheated to 200 °C. The dishes from pork (with cranberries, apricots, or prunes or plain) were baked separately for 30 min at this temperature and then at 180 °C for an additional 60 min. Ten minutes before the end of roasting, the bag was cut open from the top to brown the roast. After the dishes cooled, the fruit fillings were removed and each meat was ground separately in an electric grinder. In a dish without additives, one kilogram of raw pork tenderloin yielded 520 g of roasted meat. In the case of dishes stuffed with fruit, these amounts were 570 g for meat with apricot filling, 572 g for a dish with cranberries, and 552 g for a dish with prunes.

### 2.4. Heterocyclic Aromatic AMINES Extraction

The methodology used to isolate HAAs was previously applied in our analyses of other meat samples. It was based on the extraction and purification procedure initially developed by Gross and Grüter [34]. With some modifications, we used it to simultaneously isolate several groups of compounds from meat samples, including polar HAAs [43], nonpolar ones (carbolines) [28], PAHs [44], and their nitrogenous heterocyclic derivatives (azarenes) [28,43].

The procedure for HAA extraction involved several steps. First, a 30-g portion of each meat sample was hydrolyzed by mixing with 90 mL of a 1 mol/L sodium hydroxide solution for three hours. Six portions of 20 g each were weighed from the resulting dense suspension. Each serving corresponded to a meat content of 5 g. To obtain spiked samples, two such aliquots were spiked with five polar HAAs at 10 ng and 40 ng of each compound per g of meat by adding a standard mixture. Each of the six portions of the hydrolyzed meat sample was separately subjected to the entire extraction process. To 20 g of hydrolysate, 15 g of Extrelut and 10 mL of a 1 mol/L sodium hydroxide solution were added. The resulting thoroughly mixed mass was then transferred to a 20-mL polypropylene column. The elution of organic compounds was performed directly from Extrelut onto the SPE-PRS column using sixty mL of CH_2_Cl_2_ (with 5% toluene added). Nitrogenous heterocyclic compounds, including HAAs, were retained in the propylsulfonic acid phase. After drying the SPE-PRS column, it was washed with HCl solution (6 mL; concentration 0.01 mol/L), followed by water (2 mL), and then combined with the SPE-C18 column. Tandemly connected SPE columns (with PRS and C18 phase) were washed with ammonium acetate solution (20 mL, concentration 0.5 mol/L, pH 8). After disconnecting this tandem, each column was rinsed with water (10 mL) and dried. Heterocyclic nitrogen compounds were eluted from both columns with 4 mL of a methanol–aqueous ammonia mixture (9:1 *v*/*v*). Extracts eluted from C18-phase SPE columns contained polar HAAs (IQ, MeIQ, 8-MeIQx, 4,8-DiMeIQx), while compounds of lower polarity, including azaarenes and β- and γ-carbolines, were present in extracts from SPE-PRS columns, as shown in our previous study [28]. PhIP is classified as a polar HAA, but due to the phenyl ring in its molecule, this compound was eluted from the SPE-PRS column less effectively than imidazoquinolines and imidazoquinoxalines.

The described clean-up procedure (including hydrolysis, Extrelut, SPE-PRS, and SPE-C18 extraction steps) was performed three times for each meat sample from the four tested dishes. Due to the low content of HAAs in food, the corresponding extracts obtained from the four hydrolysates of a given meat sample, which were not enriched with standards, were combined together to produce an extract equivalent to 20 g of cooked meat. The solvents were evaporated to a volume of about 0.5 mL, with the remainder removed by a nitrogen stream. After dissolving in methanol, the fractions isolated from the SPE-PRS and SPE-C18 columns were subjected to quantitative analysis by HPLC. A quantity of 500 µL of solvent was used for spiked samples, and 200 µL was used for unenriched samples.

### 2.5. HPLC-DAD-FLD Analysis

For the chromatographic separation of HAAs, the Tosoh Bioscience (TosoHaas, Stuttgart, Germany) TSK-gel ODS 80-TM column (250 × 4.6 mm I.D.; particle size 5 µm) was used. Separations were performed under gradient conditions, using acetonitrile (A) and solution (B) containing acetonitrile (5%) and a buffer at pH = 3.3 (prepared from an aqueous solution of triethylamine and phosphoric acid (V)). The following elution program was used: solution B (100%) flowed for 2 min, followed by a linear increase (over 20 min) of acetonitrile (A) to achieve a mobile phase with 25% A and 75% B. This was followed by a linear increase (over 10 min) of acetonitrile (A) to 55% in the mobile phase. This composition was maintained for the final 10 min of the elution program. The mobile phase flow rate was 1 mL/min, and all separations were performed at 40 °C.

Determination of heterocyclic amines from the groups of imidazoquinolines (IQ; MeIQ) and imidazoquinoxalines (8-MeIQx; 4,8-DiMeIQx) involved recording the chromatogram signal areas with a DAD detector at a UV wavelength of 263 nm, chosen based on the literature data and studies conducted for standard solutions [43]. The HPLC-DAD-FLD equipment allowed recording signals corresponding to the determined compounds at several UV wavelengths, as well as fluorescence signals at selected excitation and emission wavelengths. The signal recorded for PhIP with the fluorescence detector was more intense than the corresponding signal from DAD, so in this study, PhIP was determined using the HPLC-FLD technique at excitation (λ Ex) and emission (λ Em) wavelengths of 263 nm and 400 nm, respectively. The main parameters for the detection and determination of HAAs by the HPLC-DAD-FLD method are summarized in Table 2.

### 2.6. Establishment of Basic Validation Parameters for the Determination of HAAs

Validation of the methods was aimed at determining the following parameter values: linearity range, recoveries of the determined compounds, limit of detection (LOD) and limit of quantification (LOQ), and repeatability, as well as reproducibility [45]. The LOD was determined from the ratio of the analyte signal area (S) on the chromatogram to the noise (N) signals. The LOD was set at three times the noise level (S/N = 3/1). The LOQ was defined as 3.3 times the LOD.

### 2.7. Statistical Analysis

Statistical analysis was performed using the Statistica 13.3 software (TIBCO Software Inc., Palo Alto, CA, USA). The variables were presented using basic parameters of descriptive statistics, such as mean and standard deviations. The compatibility of the variable distribution with the normal distribution was checked using Kolmogorov–Smirnov, Lilliefors, and Shapiro–Wilk tests. Comparison of the results of the HAAs analysis obtained for four meat dishes (prepared without and with three different fruits) was performed using the one-way ANOVA test and Scheffe, LSD Fisher’s, and Tukey post hoc tests. A *p* value < 0.05 was considered statistically significant.

## 3. Results

### 3.1. HAAs Concentrations Determination by HPLC-DAD-FLD

**Figure 1:**
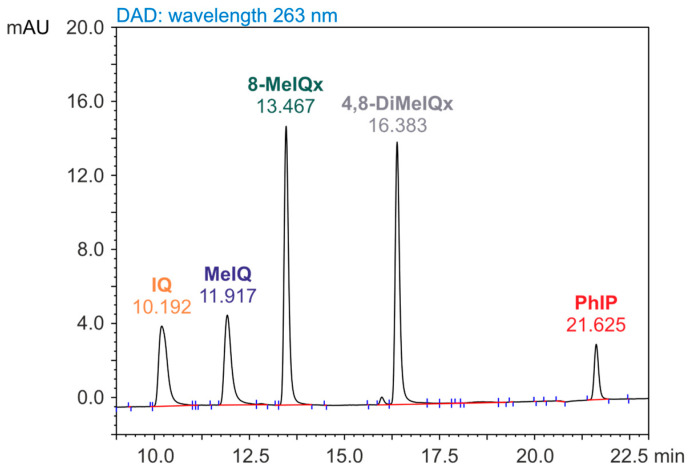
Chromatogram of HAA standard solution (concentration, 1 ng/µL; injection volume, 10 µL) recorded during analysis by the high-performance liquid chromatography with diode array detection (HPL-DAD) technique. The full names of compounds (IQ, MeIQ, 8-MeIQx, 4,8-DiMeIQx, and PhIP) are given in Table 1.

The chromatographic column used for the separation of heterocyclic aromatic amines in the standard solution contained a bed (TSK gel ODS-80TM) stable in the pH range of 2.5 to 7.5, enabling the use of a triethylamine phosphate buffer at pH = 3.3 as a mobile phase component. This type of column is commonly used for determining heterocyclic aromatic amines in food. Figure 1 shows the chromatogram obtained from the separation of the five selected HAAs solution (IQ, MeIQ, 8-MeIQx, 4,8-DiMeIQx, and PhIP), recorded by a diode array detector at the UV wavelength of 263 nm.

**Figure 2:**
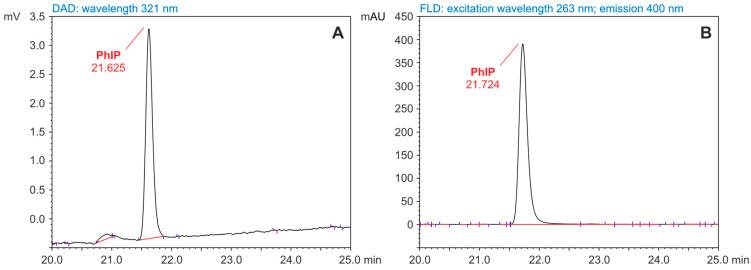
Fragments of chromatograms recorded during the analysis of PhIP standard by the high-performance liquid chromatography (HPLC) with diode array detection ((DAD) (**A**) and with fluorescence detection (FLD) (**B**) techniques (concentration, 1 ng/µL; injection volume, 10 µL).

The retention times of these compounds ranged from 10.2 min (IQ) to 21.6 min (PhIP). According to bibliographic data, PhIP could be determined using the HPLC-DAD technique [34,43,46]. As a pyridine derivative, this compound exhibited fluorescence. The chromatogram signal for PhIP recorded with FLD at excitation and emission wavelengths (λ Ex/λ Em) of 263/400 nm was characterized by a significantly higher (10-fold) intensity than the DAD signal (λ = 321 nm). For this reason, PhIP concentrations in standard solutions and fractions isolated from food samples were determined using the fluorescence detector. To illustrate the differences between HPLC-DAD and HPLC-FLD determinations of PhIP, Figure 2 shows fragments of chromatograms recorded for a standard solution of this compound.

Quantitative analysis of HAAs was conducted using the external standard method, with peak area values corresponding to each compound recorded on chromatograms by fluorescence or DAD detector. Calibration graphs were prepared by the least squares method from at least five results obtained for six calibration points. In each instance, 10 µL of the standard mixture (in methanol) was injected into the column. The linearity ranges of graphs for four HAAs (IQ, MeIQ, 8-MeIQx, and 4,8-DiMeIQx) determined by the HPLC-DAD technique ranged from 100 to 2500 ng/mL. For PhIP, determined by the HPLC-FLD technique, linearity covered a wider range: from 20 to 2500 ng/mL. The regression coefficient *r* for all calibration graphs used to calculate HAA concentrations in food samples was above 0.999. The regression coefficients *r* are presented in Table 3.

For PhIP, determined by the HPLC-FLD technique, the LOQ was 6.0 ng/mL. For other heterocyclic amines (IQ, MeIQ, 8-MeIQx, and 4,8-DiMeIQx), determined by the HPLC-DAD technique, LOQ values were ten times higher (Table 3). Each fraction isolated from food samples, dissolved in the appropriate amount of methanol, was introduced into the chromatography column at 10 µL. The calculated LOQ values for food samples ranged from 0.07 ng/g (for PhIP) to 1 ng/g of meat (IQ and MeIQ). The detection and quantification limits for the studied compounds are shown in Table 3.

To account for the potential influence of the complex food matrix on the results and determine probable compound losses, samples spiked with two HAAs (PhIP and 8-MeIQx) were tested. Meat samples enriched with standards at 10 ng/g and 40 ng/g were subjected to extraction, isolation by SPE, and determination by HPLC-FLD or HPLC-DAD techniques. The percentage recoveries were calculated using the following equation: recovery = [(C1 − C2)/C]∙100%. In this formula, C1 corresponds to the concentration of HAA in the meat sample with standards added, and C2 corresponds to the concentration of HAA in the meat sample, while C corresponds to the amount of HAA standards added to the meat. In each case, the concentration is given in ng/g. The recoveries of standards added to meat samples were 67.1% (PhIP) and 92.8 (8-MeIQx) for 10ng/g and 78.1% (PhIP) and 93.9% (8-MeIQx) for 40 ng/g, with relative standard deviations (RSDs) ranging from 5.9% to 16.2%. The results met the recovery criteria for methods determining polycyclic aromatic hydrocarbons in food. For example, per the European Union Commission’s recommendations, the recovery for carcinogenic benzo(a)pyrene should range from 50 to 120% [47].

The intra-day precision (repeatability) for HAA determination was calculated based on the standard deviations of results obtained on a single day for a series (n = 5) of 8-MeIQx and PhIP solutions with concentrations of 10 ng, 5 ng, and 1 ng (in 10 µL of methanol). Inter-day precision (reproducibility) was determined from tests on these solutions conducted over five consecutive days. Repeatability ranged from 0.27% (8-MeIQx) to 2.39% (PhIP), and reproducibility ranged from 0.93% (8-MeIQx) to 5.93% (PhIP). The obtained validation results indicated that the method used was sufficiently accurate for the determination of HAAs in meat samples.

### 3.2. Results of Concentration Determination for Heterocyclic Aromatic Amines in Meat Samples

**Figure 3:**
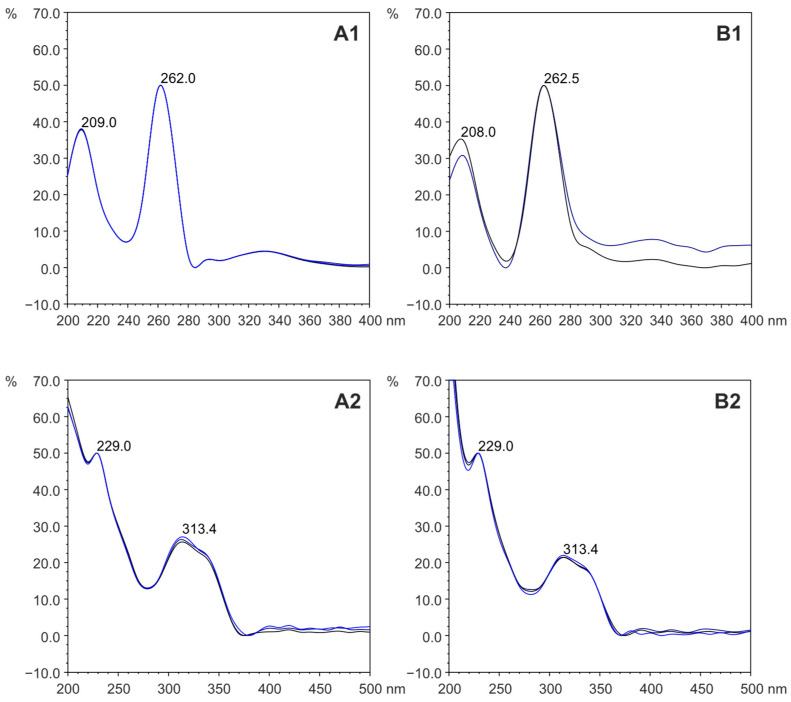
Ultraviolet (UV) spectra for standards (**A**) and compounds detected in meat samples (**B**) recorded for 8-MeIQx (**A1**,**B1**) and PhIP (**A2**,**B2**). The full names of 8-MeIQx and PhIP are given in Table 1.

Identification of HAAs in food samples involved comparing retention times of chromatogram signals recorded during HPLC-DAD-FLD analyses of fractions isolated from meat samples with times of standards and compounds detected in extracts of samples enriched with standards. Additionally, the HPLC-DAD-FLD technique allowed recording UV spectra for compounds identified as heterocyclic amines, confirming their presence in food samples through comparison with standard spectra. Examples of UV spectra for the standards 8-MeIQx and PhIP and compounds identified in food samples are shown in Figure 3.

The concentration results (in ng/g of meat) for HAAs in pork loin samples roasted in an electric oven with the use of a “foil sleeve for baking” are presented in Table 4. In extracts from the dish prepared with plain pork and from roasts stuffed with dried fruits (prunes, apricots, and cranberries), only two of the five heterocyclic amines selected for the study were detected, viz. 8-MeIQx and PhIP. Their concentrations ranged from 0.2 ng/g (PhIP, pork with cranberries) to 3.5 ng/g (8-MeIQx, plain pork). The total content of PhIP and 8-MeIQx in samples of pork loin without additives roasted in a roasting bag was 5.89 ng/g. The use of dried fruit filling reduced this concentration in dishes prepared with prunes, apricots, and cranberries by 42.3%, 46.5%, and 76.6%, respectively. The strongest inhibitory effect was observed for cranberry, which caused a reduction in PhIP concentration by almost 92%.

The 8-MeIQx concentrations were determined based on UV-DAD detector results in fractions isolated from SPE-C18 columns, while PhIP was determined using fluorescence detector results in fractions from SPE-PRS columns. The choice of the fraction for each compound was based on a comparison of HAA recoveries in meat sample extracts enriched with standards and isolated from SPE-PRS and SPE-C18 columns. In the case of PhIP, recoveries were significantly higher in extracts from the SPE-PRS column, reaching approximately 70%. PhIP recoveries in eluates from the SPE-C18 column did not exceed 35%, aligning with the results from our previous studies [43].

**Figure 4:**
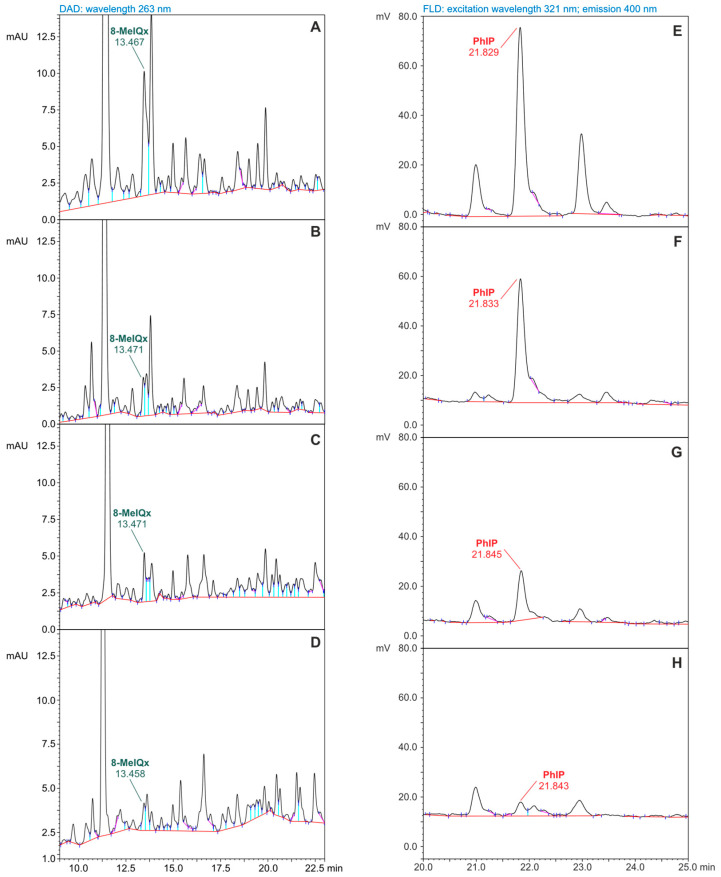
HPLC-DAD (**A**–**D**) and HPLC-FLD (**E**–**H**) chromatograms obtained during the determination of 8-MeIQx and PhIP in fractions isolated from samples of pork loin roasted (**A**,**E**) without added fruit, (**B**,**F**) with prunes, (**C**,**G**) with apricots, and (**D**,**H**) with cranberries. In each case, 10 µL from 200 µL of solution was analyzed. The full names of 8-MeIQx and PhIP are given in Table 1.

Chromatograms recorded during the determination of 8-MeIQx and PhIP in fractions isolated from meat are given in Figure 4. To illustrate HAA concentration changes due to dried fruit addition in pork loin dishes, an ordinate axis scale (indicating signal intensity) was introduced on the chromatograms, aligned with the highest peak for 8-MeIQx and PhIP (Figure 4A–D and Figure 4E–H, respectively). Each chromatogram was obtained by analyzing an equal sample amount (10 µL of 200 µL extract solution). The highest signals for 8-MeIQx and PhIP were recorded for samples from roasted pork loin without additives (Figure 4A,E). Chromatogram peaks representing these compounds were lower in extracts from roasted meat with dried fruit filling.

## 4. Discussion

### 4.1. Influence of the Preparation Method of Meat Dishes on Their Content of Heterocyclic Aromatic Amines

The selection of the five polar HAAs (IQ, MeIQ, 8-MeIQx, 4,8-DiMeIQx, and PhIP), the determination of which was the aim of our study, was based on the literature reports regarding the occurrence of these compounds in thermally treated meat, as well as their adverse health effects. HAAs that occur in fried, baked and grilled meat products in the highest concentrations were selected for our research [6,10,11,12,14,48]. In addition, four of the HAAs (IQ, MeIQ, 8-MeIQx, and PhIP) are listed by the IARC as probable or possible carcinogenic compounds [15]. They are also listed in the Report on Carcinogens issued by the US Department of Health and Human Services as reasonably projected for the group of human carcinogens [49]. People who frequently eat heat-treated meat and for whom meat, especially red meat, is the main source of protein cannot avoid exposure to HAAs. Therefore, it seems reasonable to seek and disseminate ways to heat-treat meat to reduce consumer exposure to these harmful compounds.

In the research conducted in this study, four types of pork loin dishes were prepared: one without additives and three stuffed with dried fruits (prunes, apricots, and cranberries). All dishes were baked under reproducible, identical conditions in an electric oven using a foil baking sleeve. Many factors can affect the type and content of HAAs in food. These include, foremost, the method of preparing the food, as well as the duration of the heat treatment and the rate of temperature increase [36]. Additionally, the shape and size of the heat-treated meat portion; the origin of the meat; and the presence of reducing sugars, α-amino acids, creatine, fat, and water are also factors influencing the concentration of formed HAAs [18,37,46,50]. Given the influence of such diverse factors on HAA formation, the present study used pork loin from a single animal, and aside from the dried fruits used as stuffing, all preparation and heat treatment parameters were kept identical for each dish.

Heat treatment of meat in an electric oven using a tightly sealed foil baking sleeve resembles the conditions obtained when braising meat with added water. Thus, despite the relatively long baking time (90 min), the meat remained juicy and not parched. There are limited data in the literature regarding the effect of baking meat in a foil sleeve on the formation of HAAs [51]. The results from studies described in the literature indicate that foods prepared in an electric oven tend to contain fewer HAAs than fried or grilled foods [12,18,23]. Only two compounds, out of the five HAAs designated for determination in this study, namely, 8-MeIQx and PhIP, were detected in extracts isolated from pork loin samples baked in a foil sleeve. Both of these amines have been classified by the International Agency for Research on Cancer as possibly carcinogenic to humans [15]. Their concentrations in the tested meat samples were 3.5 ng/g (8-MeIQx) and 2.4 ng/g (PhIP), respectively. According to the literature, these two amines are among the most frequently determined in thermally processed high-protein foods, likely due to their concentrations being higher than those of other compounds in this group [37,46]. The presence of PhIP and/or 8-MeIQx, with no other polar heterocyclic amines detected in food samples, has been reported in numerous research papers [12,23,37,51].

The concentrations of HAAs determined in this study for pork loin samples baked in an electric oven using a foil baking sleeve were comparable to or lower than the concentrations in pork loin dishes prepared by frying, where 8-MeIQx and PhIP were measured in ranges of 3.7 to 6.0 ng/g and 1.5 to 6.5 ng/g, respectively [43,52]. Notably, in the analyzed samples, the total concentration of only two heterocyclic aromatic amines (PhIP and 8-MeIQx) was 5.8 ng/g, which was almost equal to the total amount of four polycyclic aromatic hydrocarbons (PAHs) (benzo(a)anthracene (BaA), chrysene (Chr), benzo(a)pyrene (BaP), and benzo(b)fluoranthene (BbF)), which amounted to 6.1 ng/g in the same product [44]. The concentrations of these four PAHs, according to European Commission Regulation (EU) 2023/915 [53], should be determined in meat products (smoked and heat-treated). To date, no similar regulation has been introduced for HAAs. There are also no regulations or strategies to reduce exposure to heterocyclic amines [49]. However, according to current health recommendations published by the WHO, red meat consumption should not exceed 500 g per week for adults [1], and the Council of Europe recommends that the amount of HAAs consumed should be less than 1 µg per day [14,54].

### 4.2. Effect of Dried Fruits on HAAs Formation in Thermally Treated Meat

The available literature suggests that one way to decrease the HAA concentrations in thermally processed meat is to use antioxidant-rich additives of plant origin, in the form of spices or marinade ingredients [14,17,19,22,38,42]. Antioxidants can modify the Maillard reaction pathways that occur through a radical mechanism, thereby reducing the productivity of HAA formation reactions [21,24,25]. However, there are studies indicating an increase in the concentrations of some HAAs due to the presence of spices or marinades [12,27,28,29]. It is a tradition in Polish cuisine to prepare meat dishes baked or stuffed with various dried or fresh fruits. Nowadays, many popular cooking programs and cookbooks offer recipes that combine this old Polish tradition with modern thermal cooking methods. One such method is roasting meat in an electric oven using baking sleeves. The type of dried fruit used to stuff the pork loin and the quantity (200 g per kilogram of meat) were chosen based on recipes found in cookbooks. The results of MeIQx and PhIP determinations in pork loin roasted without additives and in fruit-stuffed meat samples (Table 4) indicated that the use of prunes, apricots, and cranberries inhibited the formation of these HAAs in meat dishes. To confirm the inhibitory effect of dried fruits on MeIQx and PhIP formation in meat dishes, we present in Figure 4 the chromatograms recorded for extracts of roasted meat samples without additives and from meats stuffed with fruits.

To date, there have been no publications in the scientific literature on the formation of HAAs in high-protein foods assessing the effect of whole fresh or dried fruits added to meat in substantial quantities, as suggested, for example, by traditional Polish recipes. The few published studies on the effects of fruit on HAA formation mainly focus on fruit extracts and their potential to reduce the concentrations of these compounds in meat products [36,37,39,40,41]. A polyphenolic-rich extract of dried apple peels introduced into ground beef at a quantity of 0.3% before frying resulted in a reduction in 8-MeIQx concentrations by 41% and PhIP concentrations by 60%. A stronger effect (reducing concentrations by 68% and 83%) was noted when the extract was applied only to the surface of the dish [39]. In another study, 3% apple extract reduced the 8-MeIQx content by 51% and PhIP by 65% [40]. Conversely, marinating beef with 0.25%, 0.5%, or 1% water-ethanol extract of berries produced dishes in which the 8-MeIQx content decreased from 47% to 100%, depending on the frying temperature and extract concentration [50]. Marinating various meats in the juices of blueberries, raspberries, and strawberries also reduced the content of amines, including PhIP and 8-MeIQx, in the fried dishes, with the strongest reduction in concentration—up to 100%—noted for dishes marinated for 12 and 24 h [37]. Pomegranate seed extract (0.5%) added to beef and poultry meat before roasting in an electric oven, frying, or grilling over charcoal resulted in lower concentrations of many HAAs, with the highest reduction observed in grilled foods, reaching 54% for 8-MeIQx and 72% for PhIP [41]. The results of the study cited above are similar to those obtained in the present study, where reductions in 8-MeIQx concentrations ranged from 31% to 67%, and PhIP concentrations ranged from 34% to 92% (Table 4). Studies confirm that plant-based additives can reduce the content of HAA group compounds, although the intensity of the changes depends on various factors, including temperature, cooking time, and the type and method of introducing the additive into the dish (marinating, spraying meat portions with the extract, or mixing it with ground meat) [23,39,40,41,42]. Some studies even indicate that there is no correlation between the antioxidant potential of additives and the degree of reduction in heterocyclic amines in meat dishes [41,55].

Dried fruits used as filling for pork loin are rich in antioxidants belonging to phenolic acids, such as gallic acid, chlorogenic acid, and ferulic acid [56]. In a study, the addition of 0.025 to 0.625 mmol of chlorogenic acid to 110 g of lamb meat roasted on a charcoal grill resulted in a reduction in many amines, including 8-MeIQx (by a maximum of 56%) and PhIP (by 100%) [55]. However, model tests using substrates for heterocyclic amines and various polyphenolic compounds did not demonstrate an inhibitory effect of chlorogenic acid on the formation of PhIP; instead, the concentration of PhIP increased almost twofold compared with a model system that did not contain this acid [57]. Polyphenols that have two hydroxyl groups in the meta position on the aromatic ring exhibit a particularly high ability to deactivate carbonyl compounds, which serve as substrates for HAAs, by forming adducts [13,58,59]. One such compound is quercetin, often cited as a factor that reduces HAA levels, especially PhIP [21,56,59]. However, some studies have reported an increase in 8-MeIQx, alongside a decrease in PhIP, under the influence of quercetin [39]. This increase in heterocyclic amines may be due to the oxidation of certain phenolic compounds, in the presence of other oxidizing agents, into quinones. These reactive carbonyl compounds can participate in the formation of HAAs from imidazoquinolines and imidazoquinoxalines through the Maillard reaction [13]. Model studies involving substrates for heterocyclic amines (such as amino acids, glucose, and creatine) and selected flavonoids, including quercetin, suggest that the mechanism behind quercetin’s inhibitory effect on PhIP formation may involve the deactivation of phenylacetaldehyde, an intermediate product in PhIP synthesis from phenylalanine [25,60]. Among the dried fruits selected as additives for pork dishes, apricots contained the highest amount of quercetin, with approximately 27 mg/100 g, while prunes and cranberries contained only small amounts (less than 1 mg/100 g) [56]. This may explain why the concentration of PhIP decreased more significantly (by 70%) in pork stuffed with apricots than the concentration of 8-MeIQx (which decreased by only 31%).

The results of HAA determinations in meat samples with dried fruits, as presented in Table 4, indicated that cranberries inhibited the formation of HAAs more strongly than apricots and prunes. Cranberries are known to contain higher levels of vitamin C compared with other fruits, along with small amounts of vitamin E [56,61]. Research by Liao et al. shows that even a small addition (0.01 to 0.1%) of vitamins C and E can reduce the concentrations of certain heterocyclic amines, including PhIP, in pork dishes [62]. Compounds with antioxidant properties can inhibit various Maillard reaction pathways. Studies confirm that they prevent the formation of HAAs by deactivating radicals that are intermediates in the synthesis of these compounds [60,63].

A review of the literature suggests that the type and content of sugar in thermally processed high-protein products are important factors determining the concentration of heterocyclic aromatic amines formed [46,64]. Studies have shown that using approximately 1% ribose and glucose in poultry meat can reduce PhIP concentrations by almost 40% [46], while fructose and glucose can reduce these concentrations by more than 40% when added to ground beef [64]. Simple sugars are not the only inhibitors of HAA formation. Studies have indicated that the addition of fructooligosaccharides at 3 g/100 g of beef also resulted in a 100% decrease in MeIQx concentration and a 66% decrease in PhIP concentration [65]. According to the literature, 100 g of dried fruits, such as apricots, cranberries, and prunes, can contain as much as 12.5 g of fructose [66]. Using 200 g of fruit as a stuffing for a 1 kg serving of pork meat could potentially increase the fructose content by approximately 2.5 g/100 g of meat compared with a dish roasted without additives. Thus, it is likely that the significant decrease in PhIP and 8-MeIQx concentrations noted in this study (Table 4) was partly due to the increased sugar concentrations in dishes prepared with dried fruit. According to Skog and Jagerstad’s theory, one possible explanation for the reduction in HAA concentrations in high-protein foods containing high sugar amounts is the conversion of sugars into 5-hydroxymethyl-2-furfural, a compound that binds to creatinine, thereby reducing the concentration of this major substrate for the synthesis of heterocyclic aromatic amines in the Maillard reaction [67].

It is challenging to clearly assess which factors most significantly influenced the reduction in PhIP and 8-MeIQx concentrations in pork dishes prepared with dried fruit. Potential contributors could include phenolic acids, other polyphenolic compounds, and antioxidant vitamins C and/or E, which may deactivate radicals that are intermediates in HAA synthesis. Additionally, the sugars present in the dried fruit could have inhibited the formation of heterocyclic aromatic amines. Importantly, these findings indicate that simple changes in meal preparation can reduce the content of mutagenic and carcinogenic compounds in meat dishes, making them healthier for consumers.

## 5. Conclusions

The applied cooking method, using dried fruits (apricots, cranberries, and prunes) as a filling for meat roasted in a roasting bag according to Polish cuisine recipes, resulted in obtaining products with a relatively low concentration of HAAs. Pork loin roasted in an electric oven in such a bag contained only two polar HAAs, PhIP and 8-MeIQx, at a total concentration of 5.9 ng/g of meat. Incorporating dried fruits as meat filling significantly reduced the total content of these compounds. The reductions in PhIP and 8-MeIQx concentrations compared with meat roasted without additives ranged from 42% for pork loin with prunes to 77% for pork loin with cranberries. The ongoing search for methods to reduce HAA levels in food underscores the need for sensitive and widely available analytical techniques to determine these compounds within complex food matrices. The procedure used for isolating HAAs is multi-step and labor-intensive, yet it effectively enables the simultaneous isolation of several groups of compounds from the complex matrix of thermally treated meat. This includes HAAs at concentrations that can be determined using the HPLC-DAD-FLD technique, which is accessible in many laboratories.

However, to summarize the research conducted, it must be stated that focusing only on the addition of three types of dried fruits and furthermore on one type of meat and one specific method of its preparation is a limitation of the study. It seems justifiable to expand the scope of this research using other fruits (dried and fresh) and different types of meats, for evaluating their effects on the formation of not only selected polar HAAs but also a greater number of compounds from the heterocyclic amine group.

## Figures and Tables

**Table 1:** Structures, names, and abbreviations of HAAs used in this studies.

Structure	Name (Abbreviation)	Structure	Name (Abbreviation)
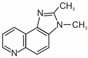	2-amino-3-methylimidazo[4,5-f]quinoline (IQ)	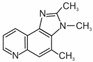	2-amino-3,4-dimethylimiazo[4,5-f]quinoline (MeIQ)
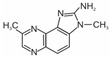	2-amino-3,8-dimethylimidazo[4,5-f]quinoxaline (8-MeIQx)	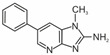	2-amino-1-methyl-6-phenylimidazo[4,5-b]pyridine (PhIP)
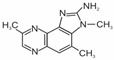	2-amino-3,4,8-trimethylimidazo[4,5-f]quinoxaline (4,8-DiMeIQx)		

**Table 2:** Main parameters of heterocyclic aromatic amines detection and determination by the HPLC-DAD-FLD method.

Parameter	Compound ^1^				
	IQ	MeIQ	8-MeIQx	4,8-DiMeIQx	PhIP
Wavelength used for HPLC-DAD ^2^ detection and determination	λ UV ^4^ = 263 nm (detection and determination)				λ UV ^4^ = 263 nm (detection)
Retention time (min)	10.192	11.197	13.467	16.383	21.625
Wavelengths used for HPLC-FLD ^3^ determination	N/A ^7^				λ Ex ^5^ = 263 nm λ Em ^6^ = 400 nm
Retention time (min)	N/A				21.724

^1^ The full names of compounds (IQ, MeIQ, 8-MeIQx, 4,8-DiMeIQx, and PhIP) are given in Table 1. ^2^ HPLC-DAD—high-performance liquid chromatography with diode array detection. ^3^ HPLC-FLD—high-performance liquid chromatography with fluorescence detection. ^4^ λ UV—ultraviolet wavelength. ^5^ λ Ex—wavelength of excitation. ^6^ λ Em—wavelength of emission. ^7^ N/A—not applicable.

**Table 3:** Data from heterocyclic aromatic amines (HAAs) determination by the HPLC technique.

HAA ^1^	Detection Method	Range of Calibration Curve (ng/mL)	Regression Coefficient *r*	LOQ ^3^ (ng/mL)	LOD ^2^ (ng/g) ^4^	LOQ ^3^ (ng/g) ^4^
IQ	UV-DAD 263 nm	100–2500	0.9999	100	0.3	1.0
MeIQ	UV-DAD 263 nm	100–2500	0.9999	100	0.3	1.0
8-MeIQx	UV-DAD 263 nm	100–2500	0.9998	60	0.2	0.7
4,8-DiMeIQx	UV-DAD 263 nm	100–2500	0.9998	60	0.2	0.7
PhIP	FLD λ Ex = 263 nm λ Em = 400 nm	20–2500	0.9999	6.0	0.02	0.07

^1^ The full names of compounds (IQ, MeIQ, 8-MeIQx, 4,8-DiMeIQx, and PhIP) are given in Table 1. ^2^ LOD—limit of detection. ^3^ LOQ—limit of quantification, for a volume of 10 µL of solution injected into the column. ^4^ ng/g of meat.

**Table 4:** Concentration ^1^ of 8-MeIQx and PhIP (ng/g meat) and the effect (%) of dried fruits on their content in pork loin roasted without and with additives.

	Concentration ^1^ (ng/g) and Inhibition (%) in Meat Samples			
Compound ^3^	Without Additives	With Prunes	With Apricots	With Cranberries
8-MeIQx	3.53 ± 0.55 ^a,2^	1.85 ± 0.25 ^b^ (47.5%)	2.44 ± 0.36 ^b,c^ (30.9%)	1.18 ± 0.16 ^b,d^ (66.6%)
PhIP	2.36 ± 0.16 ^a^	1.55 ± 0.19 ^b^ (34.3%)	0.71 ± 0.07 ^c^ (69.9%)	0.20 ± 0.015 ^d^ (91.5%)
8-MeIQx + PhIP	5.89	3.4 (42.3%)	3.15 (46.5%)	1.95 (76.6%)

^1^ Results are presented as means ± standard deviations (SDs). Each result is the average of two chromatographic measurements made for three fractions obtained by repeating the analytical procedures for isolating these compounds three times for each meat sample (n = 6). ^2^ Different letters (a to d) in a given row indicate a statistically significant difference (*p* < 0.05). ^3^ The full names of compounds (8-MeIQx and PhIP) are given in Table 1.

## Data Availability

The original contributions presented in this study are included in the article/Supplementary Materials. Further inquiries can be directed to the corresponding author.

## Supplementary Materials

The following supporting information can be downloaded at https://www.mdpi.com/article/10.3390/foods14040559/s1: No.1. Data on dried fruits used for meat stuffing.
