# Supplementary material for: Determination of Polar Heterocyclic Aromatic Amines in Meat Thermally Treated in a Roasting Bag with Dried Fruits
Source: Foods. 2025 Feb 8;14(4):559. doi: 10.3390/foods14040559 (PMC11854267; doi:10.3390/foods14040559)
Supplement: Supplementary file 1 [file foods-14-00559-s001.zip › foods-3431113-supplementary.pdf]

Supplementary Materials no. 1: Data on dried fruits used for meat stuffing

"Determination of Polar Heterocyclic Aromatic Amines in Meat Thermally Treated in a Roasting Bag with Dried Fruits"

Sylwia Bulanda, Magdalena Szumska, Agnieszka Nowak, Beata Janoszka *
and Aleksandra Damasiewicz-Bodzek

The dried fruits (prunes, cranberries and apricots) used in this study were purchased in packs of 5 x 200 g at a local store. The prunes, sourced from Polish manufacturers, contained no preservatives according to the package. The cranberries and apricots, of German origin, were preserved with sorbic acid and sulfur dioxide, respectively.
